# Analysis of the Extreme Equilibrium Conditions of an Internal Cavity Located Inside a Flat Metal Plate Subjected to an Internal Pressure *p*

**DOI:** 10.3390/ma13092043

**Published:** 2020-04-27

**Authors:** Pedro Alejandro Tamayo-Meza, Usiel Sandino Silva-Rivera, Luis Armando Flores-Herrera, Lisaura Walkiria Rodríguez-Alvarado, José Humberto Pérez-Cruz, Jesús Eduardo Rivera-López

**Affiliations:** 1 Sección de Estudios de Posgrado e Investigación, Escuela Superior de Ingeniería Mecánica y Eléctrica, U. Azcapotzalco, Instituto Politécnico Nacional, Av. Granjas No. 682, Alc. Sta. Catarina, Mexico City 02250, Mexico; jhperez@ipn.mx (J.H.P.-C.); eduardo_rivera1@hotmail.com (J.E.R.-L.); 2 Departamento de Dinámica de Sistemasy Control, Facultad de Ingeniería, Universidad Autónoma del Estado de México, Cerro de Coatepec S/N, Ciudad Universitaria, Toluca Edo. de México 50100, Mexico; usilva@convergenteng.com; 3 Departamento de Sistemas, Facultad de Ingeniería, Universidad Autónoma Metropolitana, U. Azcapotzalco, Av. San Pablo 180, Alc. Reynosa Tamaulipas, Mexico City 02200, Mexico; lwra@correo.azc.uam.mx

**Keywords:** cavity, crack formation, stress state, plate defects

## Abstract

The influence of surface bulges and cavities within metals is an important metallurgical-mechanical problem that has not been fully solved and motivates multiple discussions. This is not only related to the generation of interfaces, but also to the distribution of alloying components and elements. In this study, Laplace’s equation was used to develop a set of equations to describe these kinds of defects in plates, which arise during the development of metallurgical processes, and this can be used for the prediction of pipeline failures subjected to internal pressure. In addition, the stability conditions of a cavity under an internal pressure are analyzed. The developed method allows to identify the stress state in the generation of the cavity and its propagation. In addition to this, finite element analyses were carried out in order to show first the stress distribution around a cavity subjected to a series of theoretical operation conditions and second to show the crack growth on the tip of the cavity.

## 1. Introduction

In recent years, significant advances have been made in the development of new criteria that allows to explain the emergence, evolution, and propagation of cracks in materials. The fracture mechanics within the framework of linear and no-lineal mechanics is based on Griffith’s theory, which considers the extreme balance of stress applied in a crack or a defect of critical length [1,2,3]. Linear fracture mechanics treats a fractured body as a structure, and all the attention is focused on the small area adjacent to the tip of the crack. We denote that this theory is applicable to materials such as glass, but metals have the property of being significantly deformed in the plastic range, and considering this plasticity is a complex problem. The objective of this work is focused on establishing the complex state of stresses that arises at the tip of the crack. High strength steels are currently used for manufacturing pipelines in order to have the best safety conditions in the transport of gases and fuels [4,5]. However, serious accidents may occur during its operation if there are porosities in the material, because cavities and micro discontinuities can be generated in the material structure when it is subjected to a high internal pressure. The most probable mechanism in the formation of fractures is usually due to the deceleration of the dislocations groups in the grain boundaries in combination with the diffusion of vacancies. The micro discontinuities and micro fractures will eventually produce a crack whose propagation will cause fragmentation of the body.

The analysis of defects and cracks in metal plates, as well as their causes, has been widely studied. In some cases, hydrogen diffusion can produce embrittlement and can be associated with stress-corrosion cracking [6,7,8]. In other cases, hydrogen bubbles can appear; they are cavities below the surface of the material due to excessive internal pressure and can be the origin of hydrogen-induced cracking [9,10,11]. Hydrogen embrittlement commonly occurs in low resistance steels with magnitudes less than 80 ksi of yield strength [12], although there are also cases in aluminum [9,10,11,13].

The presence of cavities within the structure of a gas or oil pipe generates serious problems related to the combination of mechanical as well as physical and chemical fields. If the metal is used to withstand internal pressures as in transport pipelines, there are serious conditions for the formation and the growth of cavities due to the internal pressures. The aim of this study is to propose a new theoretical analysis of this failure by solving the Laplace’s equation, which is relevant since these cavities create a complex stress states that can induce fracture of the body [14,15,16,17,18,19,20,21]. Plastic deformation is developed in several stages and at multiple levels. However, the physical foundation of this process was established a few years ago. Three stages (I–III) in the crack growth process were previously identified. The transition from one stage to the other is a process based on the evolution of the dislocational structure of metal. This was demonstrated by test and studies carried out in situ at the HVTEM [22]. These studies demonstrated that the dislocational structure spontaneously reorganizes itself with dramatic alterations of the physical and mechanical properties. These are self-organizing processes because the dislocations, being defects of imbalance and energy carriers, under the action of external factors, allows the system to acquire a minimum entropy during its evolution. Because of the reconfiguration of the dislocational structure, the frontal area of a propagating fracture evolves to an extraordinary and complex structure, which with the use of the numerical simulation dramatically approaches to what actually takes place. The conditions for a crack to arise and propagates depends on the state of stresses appearing at the tip of the crack, and it is precisely at this point that the contribution of this work is focused.

## 2. Analytical Methodology

**Figure 1:**
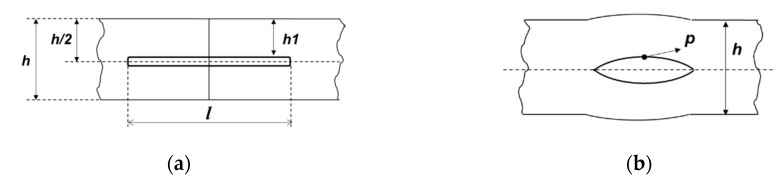
Scheme of the formation of a cavity: (**a**) Initial position of the notch before being subjected to an internal pressure load *p*; (**b**) Opening of the notch under pressure.

Consider that because of the internal pressure *p*, under which a pipeline operates, a cavity propagates and subsequently causes a fracture. Assuming that the cavity has a very small area compared to the diameter *P**_d_*** of the pipeline, it is possible to simplify the problem of the cavity critical balance through the analysis of the extreme equilibrium, which is when:

*l* ≪ *P_d_* (1)

where *l* is the length of the cavity. Additionally, we neglect the curvature of the pipeline, simplifying a problem that is originally three-dimensional. Additionally, it is assumed that the original cavity was an internal notch of circular shape with a diameter *l*. In Figure 1, the scheme of the geometry of the plate and its cavity is shown. 

**Figure 2:**
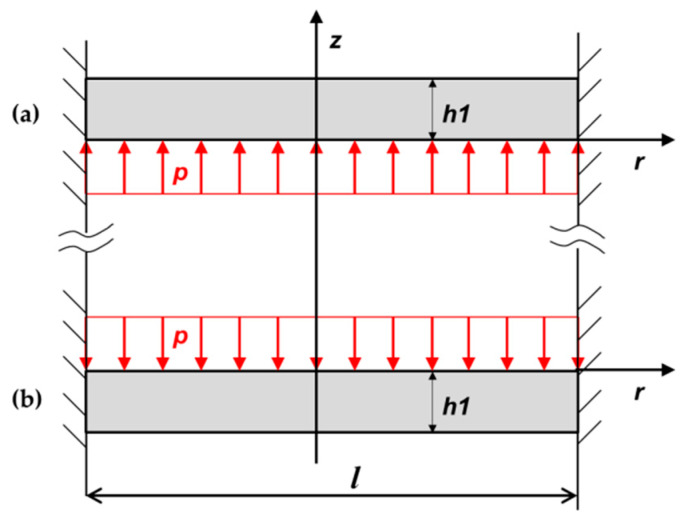
Shear-force diagram of a cavity growth inside the plate. (**a**) distributed load on the top edge and (**b**) on the bottom edge.

Without diminishing the generalities of the problem, initially it is assumed that the plane of incision of the original cavity matches with the central plane of the plate in which:

(2) $$h_{1}=\frac{h}{2}$$

where *h* is the thickness of the plate. Subsequently, when a pressure *p* is applied to the incision plane, the cavity is increased. In Figure 2a, the equivalent system 1 is shown, this means two flat plates with *h_1_* in thickness, fixed along the border and loaded with a uniformly distributed force *p*.

## 3. Selection of the Differential Segment

To analyze the evolution of the cavity as the pressure increases, the solution for a circular beam subjected to bending can be used. The known solution applicable to this problem is only possible when the load does not cause plastic deformation in the plate. The theory of elastic bending for similar plates has been previously analyzed [23,24,25,26,27]. Taking this theory for the development of the present study, we consider a load perpendicularly applied at the center of a circular cross-section, it is subsequently bended and its circular curvature is altered. This contributes to give the beam a micro-shape with a double curvature. The shape of this surface can be expressed by a radial function of the deviation as:

(3) w=f(r)

By the elastic property of the plate it is assumed that:

(4) $$w\ll h_{1}$$

In this case the bending of the beam can be analyzed independently of its strain. When the deflection *w* is comparable with the thickness *h*_1_, it will be required that the solution considers the stretching of the center of the surface.

In the theory of plate bending, two hypotheses are used to simplify the problem. First, the normal Kirchhoff invariant hypothesis, which considers that the coordinate points initially located along a normal line in the center of the plane, remains in this normal line after the deformation. Second, the hypothesis of the non-deformable layers of the plate, which considers the normal stresses negligible in comparison with the bending stresses. Considering a circular plate of constant thickness with the *Z* axis in the center, it is easy to see that the problem is symmetric, the deflection is only a function of the radial coordinates. If the angle of rotation of the normal is *θ*, then *ω* is related by:

(5) $$\theta =-\frac{d\omega }{dr}$$

**Figure 3:**
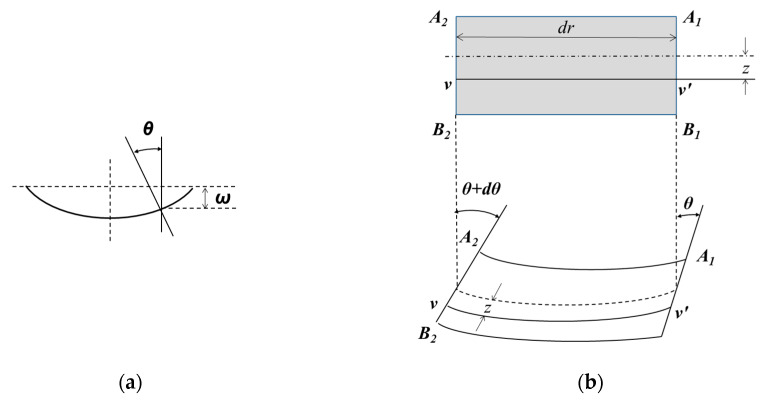
(**a**) Position of *θ* and *ω* with respect to the bending plane of the flat plate and (**b**) deflection diagram of the flat plate before and after applying the load.

The negative sign is due to the bending direction as shown in Figure 3a, with the decrement of *ω*, *θ* increases. Now, if we consider radial sections along the plate before and after the load is applied, it is noted that as a result of the plate deflection, the *A*_1_*B*_1_ normal, rotate a *θ* angle, and the *A*_2_*B*_2_ normal rotate at a *θ + dθ* angle, as shown in Figure 3b.

The *v-v’* segment, which is at a *z* distance from the center of the plane, is extended by z(θ+dθ)−z dθ=z dθ. With this, the radial elongation is:

(6) $$\varepsilon_{r}=z\frac{d\theta }{dr}$$

The radial elongation of point *v’* in the tangential direction is determined by comparing the length of the circumference in which it is located, before and after the application of the load. If, before deflection, this circumference was equal to 2πr and after the loading it becomes to 2 π(r+z dθ), then:

(7) $$\varepsilon_{t}=z\frac{\theta }{r}$$

**Figure 4:**
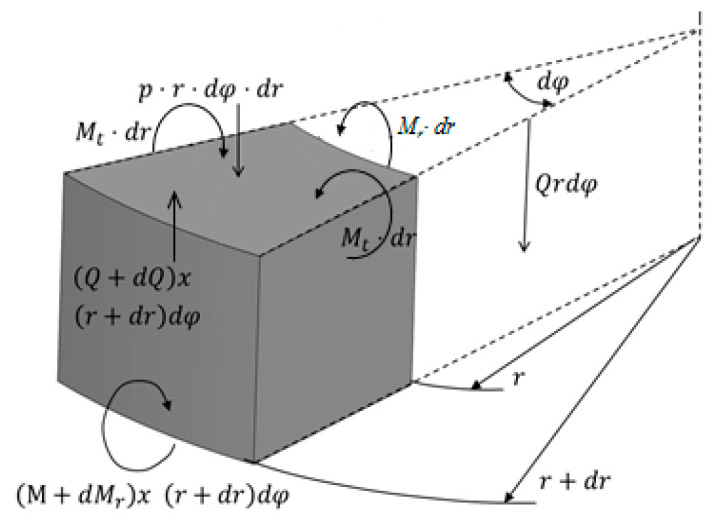
Forces and bending momentums acting on the differential element.

For a better understanding, in Figure 4 a differential plate element is shown having two radial sections, in which the angle between them is equal to dφ, and two cylindrical surfaces of r and r+dr. Now we analyze the equilibrium of this differential element after loading, replacing the effect on the plate through the internal tension. The generalized Hook’s law for our bidimensional problem is expressed by:

(8) $$\varepsilon_{r}=\frac{1}{E}\left(\sigma_{r}-\mu \;\sigma_{r}\right)$$

(9) $$\varepsilon_{t}=\frac{1}{E}\left(\sigma_{t}-\mu \;\sigma_{t}\right)$$

where *E* is the modulus of elasticity, *μ* is the Poisson’s ratio, $\sigma_{r}$ is the normal stress in the radial direction and $\sigma_{t}$ is the normal stress in tangential direction. Expressing the stress from Equations (8) and (9) and considering (6) and (7), it is possible to express the stresses in the following form:

(10) $$\sigma_{t}=\frac{E\cdot z}{1-\mu^{2}}\left(\frac{d\theta }{dr}+\mu \frac{\theta }{r}\right)$$

(11) $$\sigma_{t}=\frac{E\cdot z}{1-\mu^{2}}\left(\frac{\theta }{r}+\mu \frac{d\theta }{dr}\right)$$

The resultant forces applied on the faces of the elementary body of the plate can be determined. The tangential stresses on the *A*_1_*B*_1_ face contribute to the resulting shear load, which is parallel to the Z axis. We represent the intensity of this force, that is, the corresponding magnitude per unit length for r dθ, giving *Q* in kg. Its balance will be equal to Q r dφ. Similarly, it can be obtained for the *A*_2_*B*_2_ face, the resultant shear forces acting on this face are −(Q+dQ) (r+dr)dφ.

Since the normal forces acting on the element faces are equal in magnitude but with opposite sign, the resultant of the normal forces is equal to zero. Its effect produces only bending momentums in the vertical planes of the element. We represent the momentum intensity per unit length on the faces *A*_1_*B*_1_ and *A*_2_*B*_2_, by *M_r_* and *M_t_* in kg cm/cm. In this case, the determination of the energy balance equation is:

(12) $$M_{r}\cdot r\cdot d\varphi =r\cdot d\varphi \cdot \overset{\frac{h}{2}}{\underset{-\frac{h}{2}}{\int }}\sigma_{r}\cdot z\cdot dz$$

(13) $$M_{t}\cdot dr=dr\cdot \overset{\frac{h}{2}}{\underset{-\frac{h}{2}}{\int }}\sigma_{t}\cdot z\cdot dz$$

Using Equations (10) and (11) we obtain:

(14) $$M_{r}=\frac{E}{1-\mu^{2}}\left(\frac{d\theta }{dr}+\mu \frac{\theta }{r}\right)\overset{\frac{h}{2}}{\underset{-\frac{h}{2}}{\int }}z^{2}\cdot dz$$

(15) $$M_{t}=\frac{E}{1-\mu^{2}}\left(\frac{\theta }{r}+\mu \frac{d\theta }{dr}\right)\overset{\frac{h}{2}}{\underset{-\frac{h}{2}}{\int }}z^{2}\cdot dz$$

Since the integer of Equations (14) and (15) can be expressed in terms of $\frac{h^{2}}{12}$, then it is possible to write:

(16) $$M_{r}=D\left(\frac{d\theta }{dr}+\mu \frac{\theta }{r}\right)$$

(17) $$M_{t}=D\left(\frac{\theta }{r}+\mu \frac{d\theta }{dr}\right)$$

Thus:

(18) $$D=\frac{E\cdot h^{2}\;}{12\cdot \left(1-\mu^{2}\right)}$$

where *D* is the bending toughness of the plate. The balance equation of the selected element of the plate can be obtained by projecting the force along the *Z* axis and adding all the momentums with respect to the *Y* axis, which is tangent to the arc of radius *r* in the center of the plane, we obtain:

(19) (Q+d∅)·(r+dr)·dφ−Q·r·dφ−pr·dφ·dr=0

From which:

(20) $$p\cdot r=\frac{d}{dr}\left(Q\cdot r\right)$$

and

(21) $$\left(M_{r}+dM_{r}\right)\left(r+dr\right)d\varphi -M_{r}rd\varphi -prd\varphi dr\frac{dr}{2}-M_{t}drd\varphi +\left(Q+dQ\right)\left(r+dr\right)d\varphi dr=0$$

By neglecting higher order components, the Equation (21) can be expressed as:

(22) $$M_{t}-\frac{d}{dr}\left(M_{r}\cdot r\right)=Q\cdot r$$

The remaining balance equations satisfy the identities by the symmetry conditions. Setting up Equation (18) into Equation (22), under the condition that *D* is constant, yields to:

(23) $$r\frac{d^{2}\theta }{dr^{2}}+\frac{d\theta }{dr}-\frac{\theta }{r}=-\frac{Qr}{D}$$

Now we rearrange as follows:

(24) $$\frac{d}{dr}\left[\frac{1}{r}\cdot \frac{d\left(\theta r\right)}{dr}\right]=-\frac{Q}{D}$$

Next, we integrate twice to obtain the radial distribution of the rotation angles:

(25) $$\theta =C_{1}r+\frac{C_{2}}{r}-\frac{1}{D\cdot r}\int^{\text{​}}\left[r\int^{\text{​}}Qdr\right]dr$$

The integration constants *C*_1_ and *C*_2_, are determined from the boundary conditions for each specific case. Equation (6) is used to determine the radial function of the deflection, and using Equation (12), the momentum diagrams can be obtained according to Equations (14) and (15). From Equations (10), (11), and (18), the functions $\sigma_{r}=f\left(M_{r}\right)$ and $\sigma_{t}=f\left(M_{t}\right)$ are:

(26) $$\sigma_{r}=\frac{E\cdot z}{1-\mu^{2}}\cdot \frac{M_{r}}{D}$$

(27) $$\sigma_{t}=\frac{E\cdot z}{1-\mu^{2}}\cdot \frac{M_{t}}{D}$$

Substituting the expression for the toughness of the plate:

(28) $$\sigma_{r}=\frac{12M_{r}}{h^{2}}\cdot Z$$

(29) $$\sigma_{t}=\frac{12M_{t}}{h^{2}}\cdot Z$$

In this case, the maximum stress values are achieved on the surfaces of the plate, when $\pm \frac{h}{2}$:

(30) $$\sigma_{r}^{max}=\pm \frac{6M_{r}}{h^{2}}$$

(31) $$\sigma_{t}^{max}=\pm \frac{6M_{t}}{h^{2}}$$

## 4. On the Curvature of the Cavity

Considering the problem as symmetrical with respect to the center of the plane, we analyze just the lower part. Later we analyze when the convexity is formed in the upper side. It can be considered that the initial position of the plate corresponds to the diagram in Figure 2. We can determine *Q*(*r*) from the equilibrium conditions for a round plate element, which was concentrically sectioned:

(32) $$Q\left(r\right)\cdot 2\pi \cdot r=p\cdot \pi \cdot r^{2}$$

(33) $$Q\left(r\right)=\frac{p\cdot r}{2}$$

So, if we substitute Equations (33) and (34) into (24), and integrate twice to obtain:

(34) $$\begin{array}{c} \theta =C_{1}\cdot r+\frac{C_{2}}{r}-\frac{1}{D\cdot r}\int^{\text{​}}\left[r\int^{\text{​}}\frac{p\cdot r}{2}dr\right]\\ =C_{1}\cdot r+\frac{C_{2}}{r}-\frac{1}{D\cdot r}\cdot \frac{p\cdot r^{4}}{16}\\ =C_{1}\cdot r+\frac{C_{2}}{r}-\frac{p\cdot r^{3}}{D\cdot 16} \end{array}$$

In this case r=0, in the center of the plate where θ=0, it follows that $C_{2}=0$

(35) $$\theta =C_{1}\cdot r-\frac{p\cdot r^{3}}{16D}$$

For a fixed contour θ(r)=0, we obtain:

(36) $$C_{1}=\frac{p\cdot R^{2}}{16D}$$

Finally, the Equation (36) can be rewritten as:

(37) $$Q=\frac{p}{16D}\left(R^{2}\cdot r-r^{3}\right)$$

Using Equation (6), we can see that:

(38) $$\begin{array}{c} d\omega =-Qdr\\ \omega =-\int^{\text{​}}\frac{p}{16D}\left(R^{2}\cdot r-r^{3}\right)dr\\ \omega =\frac{p}{16D}\cdot \left\{C_{3}-\frac{1}{2}R^{2}r^{2}+\frac{r^{4}}{4}\right\} \end{array}$$

Now, ω(R)=0 in order that:

(39) $$C_{3}=\frac{1}{4}R^{4}$$

Therefore,

(40) $$\omega =\frac{p}{16D}{\left(R^{2}-r^{2}\right)}^{2}$$

From Equation (18) we find the expressions for $M_{r}\left(r\right)$ and $M_{t}\left(r\right)$:

(41) $$M_{r}=\frac{p}{16}\left[R^{2}\left(1+\mu \right)-r^{2}\left(3+\mu \right)\right]$$

(42) $$M_{t}=\frac{p}{16}\left[R^{2}\left(1+\mu \right)-r^{2}\left(1+3\mu \right)\right]$$

**Figure 5:**
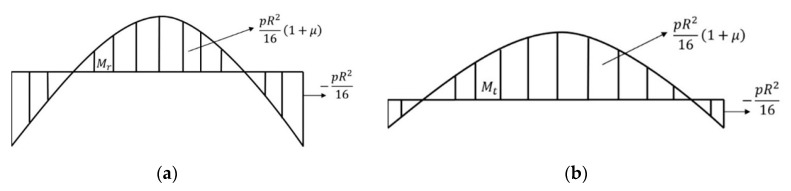
Bending-momentum diagrams of the system for (**a**) radial and (**b**) tangential.

In Figure 5, the momentum diagrams are shown, they are calculated with the use of these expressions:

(43) $$r=0;\;\;M_{t}=\frac{pR^{2}}{16}\left(1+\mu \right)$$

(44) $$r=R;\;\;M_{t}=\frac{pR^{2}}{16}\left(1+\mu -3-3\mu \right)$$

(45) $$r=0;\;\;M_{r}=\frac{pR^{2}}{16}\left(1+\mu \right)$$

(46) $$r=R;\;\;M_{r}=\frac{pR^{2}}{16}\cdot 2$$

The maximum stresses are reached in the fixed contour from the internal side of the cavity. We analyze the stress of the plate at the fixed support:

(47) $$\sigma_{1}=\sigma_{r}=\frac{3pR^{2}}{4h^{2}}$$

(48) $$\sigma_{2}=\sigma_{t}=\frac{3\mu \cdot p\cdot R^{2}}{4h^{2}}$$

(49) $$\sigma_{3}=0$$

The equivalent stresses are:

(50) $$\sigma_{eqv}=\sigma_{1}-k\cdot \sigma_{3}=\frac{3\cdot p\cdot R^{3}}{4h^{2}}$$

The maximum deviation is reached in the center of the plate for *r* = 0.

(51) $$\omega =\frac{pR^{4}}{16D}$$

The stress state in the plate center is more adverse in the outside surface of the cavity, in which the tensile stresses are:

(52) $$\sigma_{1}=\sigma_{r}=\frac{p\cdot R^{2}\left(1+\mu \right)6}{16h^{2}}=\frac{3p\cdot R^{2}\left(1+\mu \right)}{8h^{2}}$$

(53) $$\sigma_{2}=\sigma_{t}=\frac{p\cdot R^{2}\left(1+\mu \right)6}{16h^{2}}=\frac{3p\cdot R^{2}}{8h^{2}}\left(1+\mu \right)$$

(54) $$\sigma_{3}=0$$

(55) $$\sigma_{equiv}=\frac{3p\cdot R^{2}\left(1+\mu \right)}{8h^{2}}$$

So far, we have analyzed the formation of cavities, without taking into account that the pipeline is exposed to an internal pressure *p*_0_. In approximation with the theory of membranes, from the Laplace’s equation it is possible to calculate the *σ_m_* and *σ_t_* stresses obtained under a *p*_0_ pressure:

(56) $$\frac{\sigma_{m}}{R_{m}}+\frac{\sigma_{t}}{R_{t}}=\frac{p_{0}}{h}$$

where *σ_m_* is the stress in the meridional direction of the pipeline, *σ_t_* is the stress in the tangential direction, *R_m_* is the radius of curvature in the meridional direction, and *R_t_* is the radius of curvature in the tangential direction of the pipeline. For a pipeline of $R_{m}=\infty$

(57) $$\sigma_{t}=\frac{p_{0}\cdot R\cdot t}{h}$$

(58) $$\sigma_{t}=\frac{p_{0}\cdot D}{2h}$$

Analyzing the balance of the pipeline in the axial direction, we can express:

(59) $$p_{0}\cdot \pi \frac{D^{2}}{4}=\sigma_{m}\cdot \pi \cdot D\cdot h$$

From which we obtain:

(60) $$\sigma_{m}=\frac{p_{0}\cdot D}{4h}$$

**Figure 6:**
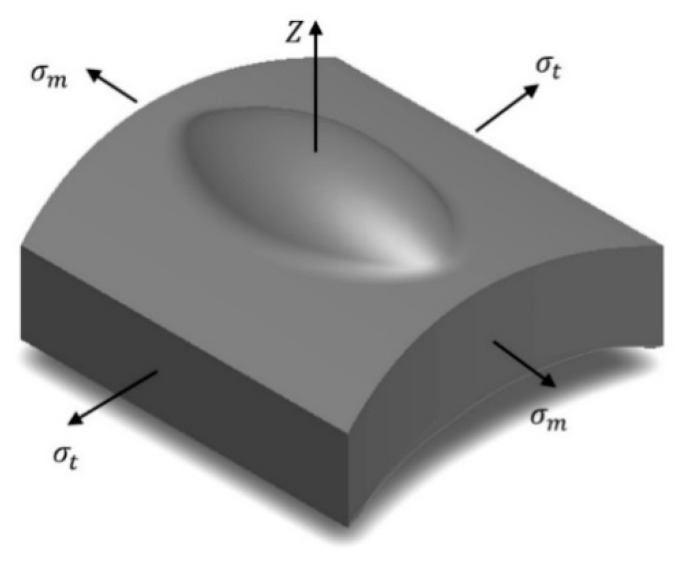
Scheme of stress distribution in a cavity on a flat plate.

Therefore, the selected differential element of the pipeline with a bulge or a cavity is shown in Figure 6. In this approach there are no stresses in the normal direction to the pipeline surface.

Therefore, in the contours of the plate that form the cavity, the stresses arising from the pressure in the duct and the pressure inside the cavity must be added. Analyzing only the axial direction:

(61) $$\sigma_{m}+\sigma_{r}=\frac{p_{0}\cdot D}{4h}+\frac{3p\cdot R^{2}}{4h^{2}}=\sigma_{1}$$

(62) $$\sigma_{2}=\frac{3}{4}\cdot \mu \cdot \frac{p\cdot R^{2}}{h^{2}}+\frac{p_{0}\cdot D}{2h}$$

and in the tangential direction:

(63) $$\sigma_{3}=0$$

To analyze the axial section of the pipe, we separate the portion of the plate that limits the cavity, which is under a *p* pressure and we have replaced its effect by an internal stress distribution in the contour. Thus, we can analyze the axial slide of the pipeline with a cavity as a plate with a crack. It is also considered that its contour is under a uniformly distributed stress and momentum. The probability of this crack to propagate should be analyzed within the background of fracture mechanics. Even before $\sigma_{1}=\sigma_{m}+\sigma_{r\;max}$, which is the yield strength from the elastic locations and after the increment in the yield point in the elastic-plastic range $\sigma_{1}>\sigma_{0.2}$. The problem can be analyzed in the plastic range, until all the segments of the duct that surround the cavity are under a *p* pressure.

**Figure 7:**
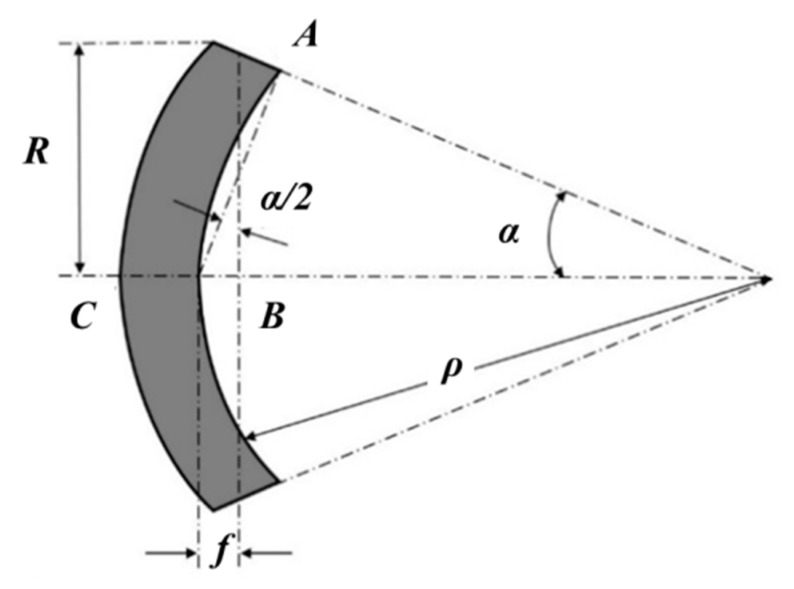
Scheme of the flat plate after being subjected to the load.

The behavior of the material enclosing the cavity under this pressure after its transition to a plastic state requires a special analysis. To solve this problem, we apply an approach developed by Feodosiev [28] to describe the bending of a membrane. First, we assume that the stress is uniformly distributed in the plate thickness, and the surface of the plate acquires a spherical shape. In Figure 7, the geometry of the plate after being subjected to the load is shown.

Considering that ρ is the radius of curvature of the plate after plastic bending, we get:

(64) $$\rho =\frac{R}{sen\alpha }$$

Since α is very small, we can assume that:

(65) $$\rho \approx \frac{R}{\alpha }$$

The deflection of the plate *f*, is:

(66) $$f=R\;tg\frac{\alpha }{2}\approx \frac{R\alpha }{2}$$

and the meridional and tangential stresses in the plate are:

(67) $$\sigma_{m}=\sigma_{t}=\frac{p\rho }{2h}$$

From the Laplace’s equation $\rho =\frac{R^{2}}{2f}$, thus:

(68) $$\sigma_{m}=\sigma_{t}=\frac{pR^{2}}{4hf}$$

Furthermore, using relationships based on the theory of plasticity, the axis coordinates are oriented so that $\sigma_{z}=0,\;\sigma_{x}=\sigma_{m},\;\sigma_{y}=\sigma_{t}$, thus:

(69) $$\varepsilon_{m}=\frac{\varepsilon_{i}}{\sigma_{i}}\;\left(\sigma_{m}-\frac{1}{2}\;\sigma_{t}\right);\;\varepsilon_{t}=\frac{\varepsilon_{i}}{\sigma_{i}}\left(\sigma_{t}-\frac{1}{2}\sigma_{m}\right)$$

From which $\sigma_{m}$ and $\sigma_{t}$,

(70) $$\sigma_{m}=\frac{4}{3}\frac{\sigma_{i}}{\varepsilon_{i}}\left(\varepsilon_{m}+\frac{1}{2}\varepsilon_{t}\right);\;\;\;\;\;\sigma_{t}=\frac{4}{3}\frac{\sigma_{i}}{\varepsilon_{i}}\left(\varepsilon_{t}+\frac{1}{2}\varepsilon_{m}\right)$$

Substituting into equation of plasticity:

(71) $$\begin{array}{c} \varepsilon_{z}=\frac{\varepsilon_{i}}{\sigma_{i}}(0-\frac{1}{2}\left[\frac{4}{3}\frac{\sigma_{i}}{\varepsilon_{i}}\left(\varepsilon_{m}+\frac{1}{2}\varepsilon_{t}\right)+\frac{4}{3}\frac{\sigma_{i}}{\varepsilon_{i}}\left(\varepsilon_{t}+\frac{1}{2}\varepsilon_{m}\right)\right]=-\frac{2}{3}\varepsilon_{m}-\frac{1}{3}\varepsilon_{t}-\frac{2}{3}\varepsilon_{t}-\frac{1}{3}\varepsilon_{m}\\ =-\left(\varepsilon_{m}+\varepsilon_{t}\right) \end{array}$$

Now, substituting Equation (66) into the equation for the strain intensity, we obtain:

(72) $$\varepsilon_{i}=\frac{2}{\sqrt{3}}\sqrt{\varepsilon_{m}^{2}+\varepsilon_{m}\varepsilon_{t}+\varepsilon_{t}^{2}}$$

Since $\varepsilon_{m}=\varepsilon_{t}=\varepsilon$

(73) $$\varepsilon_{i}=2\varepsilon$$

The elongation of the plate after bending in the plastic range can be determined by the difference in the length of the *AC* arc and the *AB* line:

(74) $$\varepsilon =\frac{\rho \alpha -\rho \;sen\alpha }{\rho \;sen\alpha }\approx \frac{\alpha^{2}}{6}=\frac{2f^{2}}{3R^{2}}$$

Locating Equation (68) into Equation (67’) we obtain:

(75) $$\varepsilon_{i}=\frac{4}{3}\frac{f^{2}}{R^{2}}$$

To calculate the intensity of the stress *σ_i_*, considering that *σ_z_ =* 0, and *σ_m_ = σ_t_*

(76) $$\sigma_{i}=\frac{\sqrt{2}}{2}\sqrt{\sigma_{m}^{2}+\sigma_{m}^{2}}$$

and

(77) $$\sigma_{m}=\frac{pR^{2}}{4hf}$$

Equations (71) and (69) can be used to obtain the function f=f(p). To achieve this, we give a value for the deflection *f*, calculate *ε_i_* with the Equation (69), and by using the stress–strain curve in coordinates *σ_i_*–*ε_i_* we determine *σ_i_*. Then, using Equation (71) we calculate the pressure. Knowing the values of *f* and *p* we graph the curve. Also, it may be employed the tensile curve expressed in parabolic coordinates as σ0+Kεi12.

## 5. Results and Discussion

Two cases were analyzed by using the Academic version of the ANSYS^®^ software (2020 R1) in order to illustrate the mechanical behavior around the cavity, a group of numerical analysis with individual CAD models were created for each case. The first one is divided into three subcases: (a) to (c). The purpose of these computational finite element analyses was to identify the deformations of the cavity in the horizontal and vertical axis. The second case is a qualitative analysis of fracture propagation caused in a small portion of the cavity, specifically in the tip of the crack for a case (a). In case (b), the propagation of the crack is caused by a pressure load applied on the internal faces of the tip crack. The material properties of the API X80 with a Yield Strength of 555MPa were considered for the analysis [29]. The analyses of the first stage must be observed as a group of instantaneous static moments in which the stresses can change according to the applied boundary conditions described below and the second stage was calculated with the fracture tool of the academic ANSYS^®^ software.

### 5.1. First Stage

**Figure 8:**
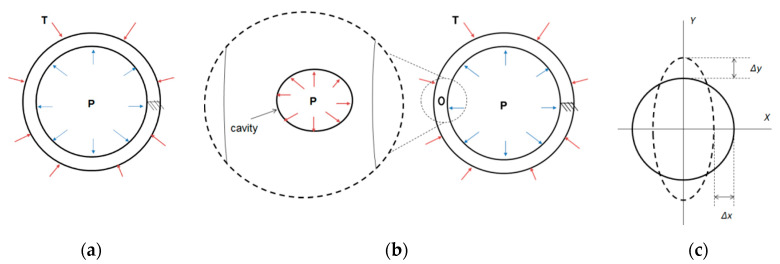
(**a**) Load conditions applied in the first case, (**b**) location of the cavity with internal pressure and (**c**) differences in the horizontal and vertical displacements.

A commercial tube with 20 inch in diameter and 0.5 inch (0.0127 m) in thickness was modelled for this stage with the following boundary conditions: Case 1a considers three different external load temperatures; T1 = −30 °C, T2 = 22 °C, and T3 = 50 °C applied on the external face of the tube to create contraction or dilatation and the solution is carried out with the ANSYS^®^ 2020 R1 Thermal analysis module. The results of this analysis are loaded in the static structural module to include the effect of the applied pressure creating a thermal-structural coupled field analysis. For each selected temperature, the tube is subjected to an internal pressure P of 10, 90 and 180 PSI as shown in Figure 8a. The right side of the tube was restricted in such a way that the coordinate displacements are: *x* = 0, *y* = 0 and *z* = 0. Case 1b, considers also the mentioned operation conditions of temperature and pressure as in the previous case plus the presence of a 200µm cavity located on the opposite side of the displacement restriction as shown in Figure 8b. This case considers the same pressure magnitude applied inside the cavity (p_c_) and the tube (p_t_). Case 1c, considers the same model of previous case without the presence of the pressure in the tube, p_t_ is eliminated. The model required sliced areas to create results paths, the paths were used to read the results always in the same nodes for the *x* and *y* coordinates as shown in Figure 8c.

**Figure 9:**
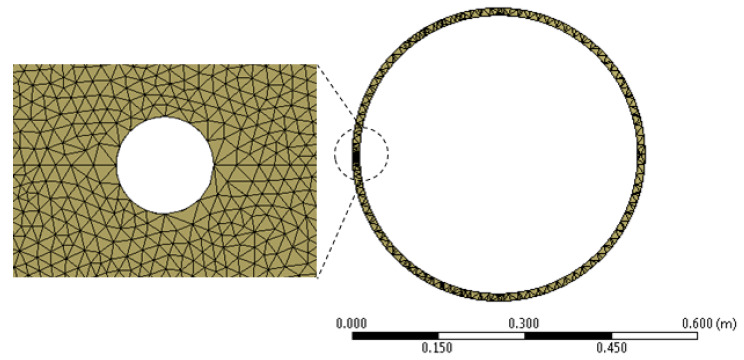
Resulting mesh around the cavity.

Tetrahedron type elements were selected to construct the continuum, Figure 9 shows the resulting mesh for the tube model and a close view of the refined mesh around the cavity, the creation of the meshing required the use of various size controls for the edges, areas and volumes.

**Figure 10:**
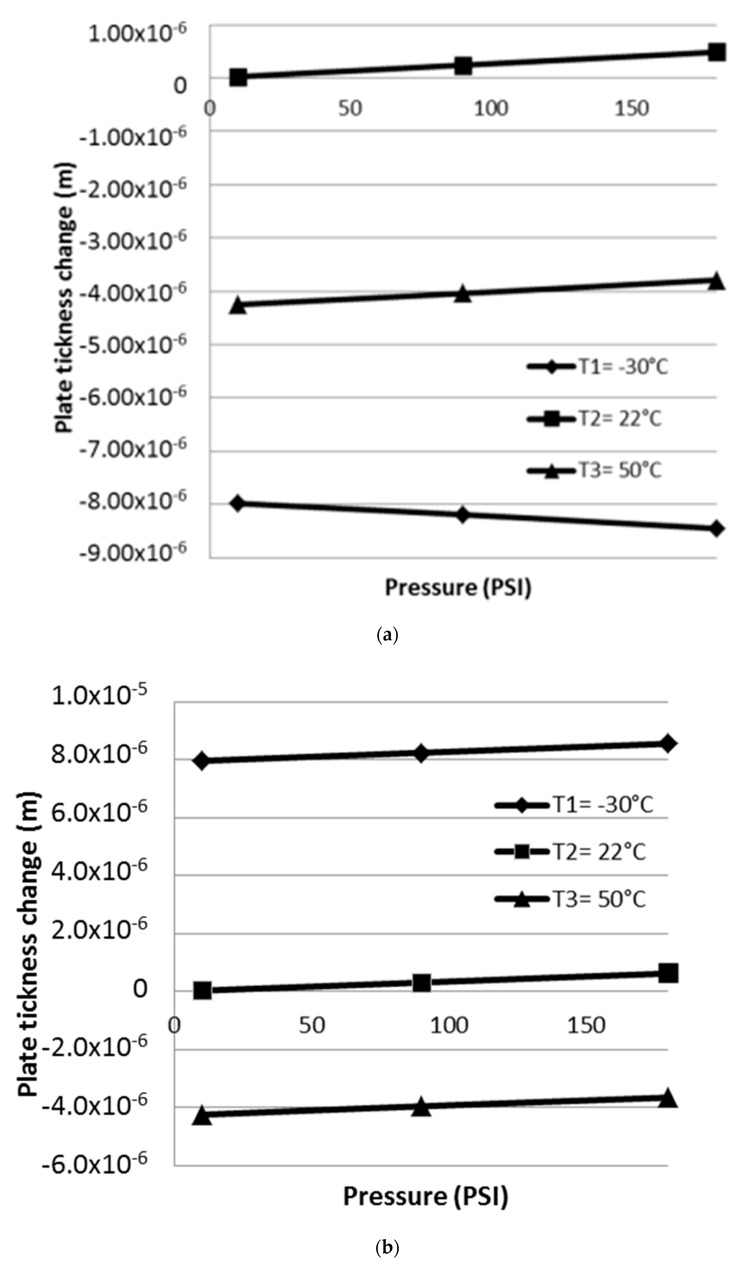

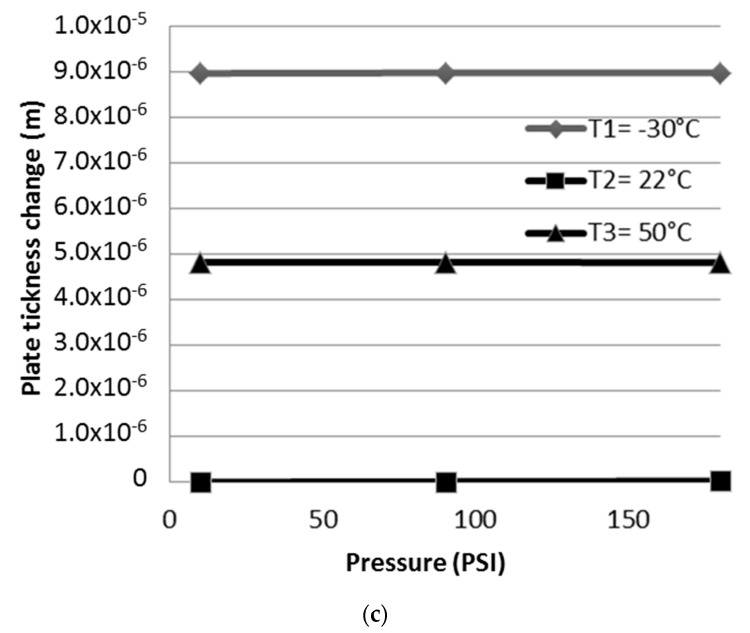
Changes in tube thickness for each case, in (**a**) tube without cavity, (**b**) tube with cavity and with pressure in p_c_ and p_t_, and (**c**) tube with cavity, pressure in p_c_ and p_t_ is eliminated.

The selection of the cases comes from the fact that in real operation, tubes are subjected to a series of complex conditions like external temperature changes depending on the geographic location. With respect to the pressure changes, they were considered because of the flow rate variations caused for example during maintenance operations. Many other real conditions like tube inclination, friction loss, internal liquid reactions, etc., are no considered in this study. As a result, Figure 10 shows the plate thickness changes with maximum values found at T1 = −30 °C.

**Figure 11:**
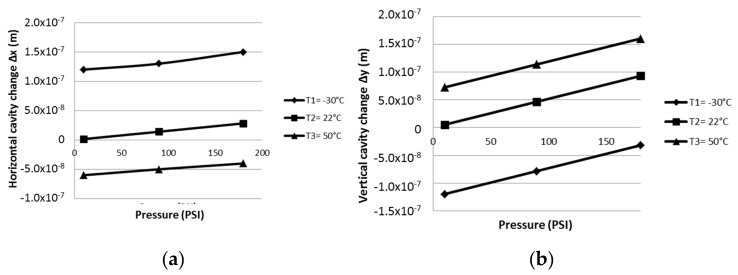

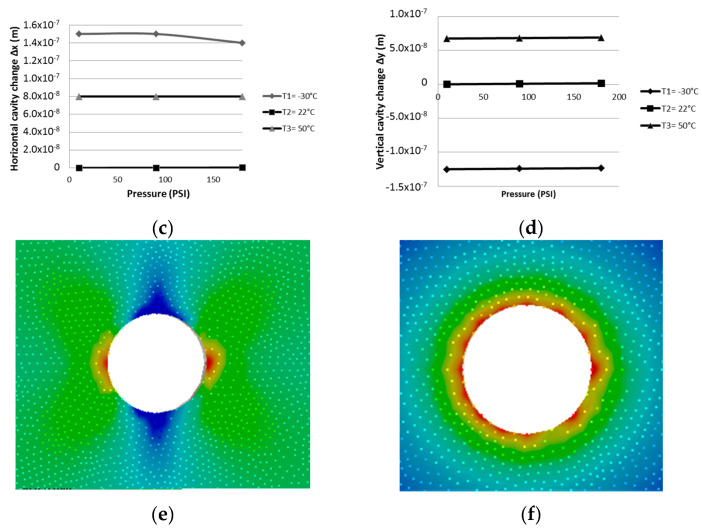
Dimensional differences with the presence of p_c_ and p_t_ in (**a**) the horizontal side of the cavity (Δx), (**b**) the vertical side (Δy), (**c**) Δx when p_t_ is eliminated, (**d**) Δy when p_t_ is eliminated, (**e**) the stress distribution around the cavity when p_c_ and p_t_ is present and (**f**) the stress distribution around the cavity when p_t_ is eliminated.

The cavity inside the plate subjected to different temperatures and pressures, suffers dimension changes which are shown in Figure 11a,b. Δ*x* and Δ*y* obtained with the presence of p_c_ and p_t_. Figure 11c,d, shows the resulting Δ*x* and Δ*y* obtained when p_t_ is eliminated. With respect to the stress distribution, the presence of the pressure inside the tube creates a difference around the cavity as shown in Figure 11e. The maximum stress levels are located on the horizontal sides and the magnitude depends on the applied pressure. Figure 11f, shows the stress distribution around the cavity without the pressure inside the tube.

### 5.2. Second Stage

**Figure 12:**
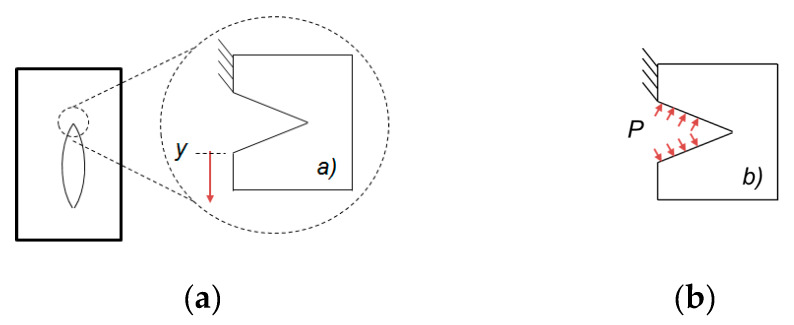
Load conditions applied for the second case, in (**a**) displacement of the material in the vertical direction and (**b**) internal pressure applied inside the cavity.

Figure 12a, shows the portion of the cavity selected for a different analysis in which a new CAD file was created. The purpose of these analyses is to observe the qualitative difference of crack propagation created by (a) the displacement of the material in the vertical direction and compare with the crack propagation caused by (b) the internal pressure in the cavity as shown in Figure 12b. The upper vertical side of the model was restricted in *x* = 0, *y* = 0 and *z* = 0.

**Figure 13:**
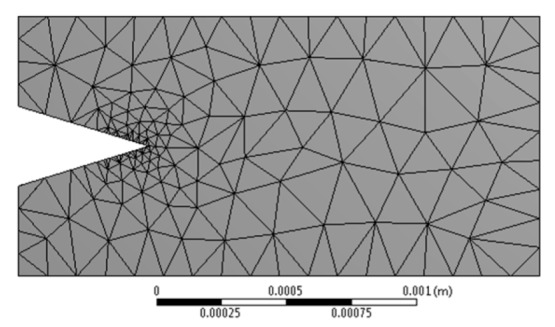
Pre-meshed crack for the second case.

The resulting tetrahedral mesh for the second case is shown in Figure 13. The model must be observed as a pre-meshed crack, this means that the model considers a generated tip from which the fracture can propagate. It is possible to observe the mesh size changes as a consequence of the sphere of influence located on the tip of the crack.

**Figure 14:**
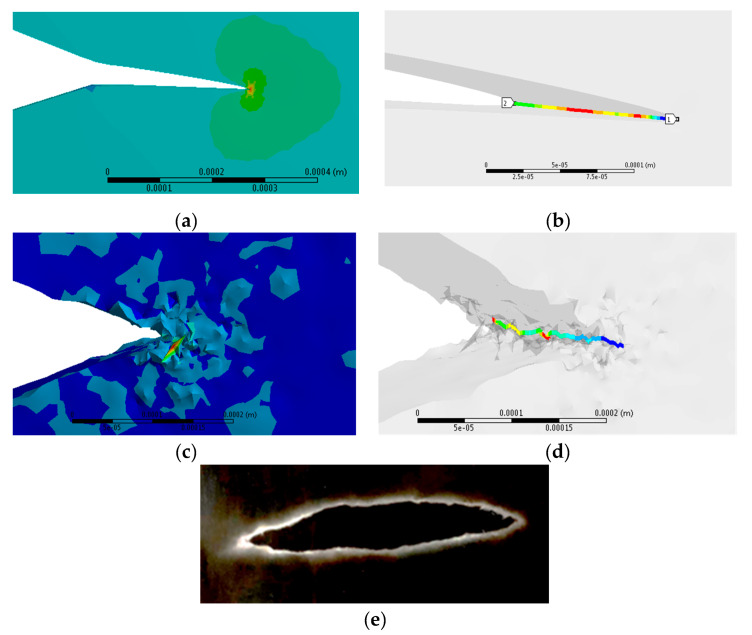
(**a**) Stress distribution around the tip of the crack in case 2a; (**b**) the horizontal line along the tip of the crack in case 2a; (**c**) Stress distribution around the tip of the crack in case 2b; (**d**) the erratic line along the tip of the crack created in case 2b and; (**e**) example of an evolved cavity observed in a HTVEM at ×50,000 in a molybdenum single crystal.

Figure 14a, shows the stress distribution created during the propagation of the crack for case 2a, a straight propagation is observed with a slight downward inclination, which is the same direction of the initial displacement. The maximum stress level is maintained at the front of the crack and the permanence of a horizontal line across the width of the crack growth can be observed as shown in Figure 14b, which has a small rotation with respect to the vertical axis to show the mentioned line. Figure 14c, shows the final geometry obtained from the crack growth, created by the pressure on the internal walls. Now the pressure generates a random break on the tip of the crack, both in direction and in magnitude, it is observed the creation of instantaneous areas during that process with different normal planes from those of the original geometry. Figure 14d, shows that the horizontal line along the tip of the crack no longer remains uniform on any of the *x*, *y* and *z* coordinate axes. Figure 14e, shows an example of micrograph of a similar crack growth with 200µm in length produced by internal pressure. It was taken in a High Voltage Transmission Electron Microscope (HTVEM), the random nature of the crack growth is observed.

## 6. Conclusions

The equilibrium and growth of a cavity inside a pipeline was analyzed, considering the effect of the internal pressure under which the pipeline works. The problem was considered as bidimensional for a cavity of the same characteristics but located inside a flat plate and was solved using the theory of elastic bending. Subsequently by using the Laplace equation, the increased stresses in the meridional and tangential directions of the pipe under its internal pressure were determined. Consequently, an equation to determine the behavior of the cavity in the flat plate in the plastic range was also obtained. A sudden increment in the stress levels was observed in the tip of the crack in comparison with the initial stage of cavity formation. It can be also observed that the appearance of maximum stress levels, instantaneous formation of small portion areas, and crack growth caused by pressure increments follow a random pattern which complicates the prediction of the future trajectory.
